# Brain Stimulation and Neuroplasticity—Series II

**DOI:** 10.3390/brainsci12081084

**Published:** 2022-08-16

**Authors:** Ulrich Palm, Samar S. Ayache, Moussa A. Chalah

**Affiliations:** 1 Department of Psychiatry and Psychotherapy, Klinikum der Universität München, 80336 Munich, Germany; 2 Medical Park Chiemseeblick, 83233 Bernau-Felden, Germany; 3 EA4391 Excitabilité Nerveuse et Thérapeutique, Université Paris-Est-Créteil, 94000 Creteil, France; 4 Service de Physiologie—Explorations Fonctionnelles, Hôpital Henri Mondor, Assistance Publique—Hôpitaux de Paris, 94000 Creteil, France

## 1. Introduction

Following the great success of the first series of the Special Issue “Brain Stimulation and Neuroplasticity” [1], this second series is once again dedicated to collecting a variety of high-quality research articles on different brain stimulation techniques and related interventions as well as their impact on neurobiological, neurophysiological, and clinical outcomes.

In this editorial, the articles included in this Special Issue are highlighted regarding their impact on the field. Therefore, papers are grouped into work covering a neurophysiological point of view, reflecting the use of neurobiological or neurophysiological parameters, predictors, or outcomes in brain stimulation or related methods in healthy volunteers or patients, or into work covering a clinical point of view, addressing the use of simulation methods for the improvement of neuropsychiatric disorders. The studies published in this Special Issue include work on central and peripheral nervous system stimulation techniques involving electrical and magnetic stimulation protocols.

## 2. Neurophysiology and Neurostimulation

The neurophysiological studies included in this Special Issue involve work implementing central and peripheral stimulation techniques.

Beginning with central stimulation, a study by Hosel and Tremblay [2] presented a neurophysiological investigation on the modulation of motor-evoked potentials (MEPs) in 19 healthy individuals using a modified version of intermittent theta burst stimulation (iTBS), which is a special form of repetitive transcranial magnetic stimulation (rTMS). MEP facilitation was observed in 68.42% of cases following a single session of 30 Hz/6 Hz iTBS, suggesting the neuromodulatory potential of this paradigm. In addition, the recruitment of early indirect waves (I-waves) appeared to predict MEP response following iTBS, which might serve as a neurophysiological predictor of response if replicated in upcoming controlled works.

In addition, Psomiades et al. [3] applied up to 20 sessions of bitemporal or right unilateral ECT in a clinical study involving 23 patients with treatment-resistant depression (brief or ultra-brief pulse stimulation (1 or 0.3 ms, respectively); number of sessions based on individual clinical observations). They showed that patients with higher mature Brain-Derived Neurotrophic Factor (mBDNF) levels before electroconvulsive therapy had a better clinical outcome (remission based on the Montgomery–Åsberg Depression Rating scale <10). Therefore, mBDNF—which plays a role in neural plasticity and neural network survival—could be a marker for clinical treatment decisions.

Moreover, Suzuki et al. [4] investigated motor cortex stimulation using different transcranial alternating current (tACS) protocols (alpha-tACS at 10 Hz vs. beta-tACS at 20 Hz vs. sham) in a randomized sham-controlled crossover study involving 16 healthy individuals. After testing several setups, they adopted the Cz-CP1 setup (according to the electroencephalographic (EEG) system for electrode positioning), which appeared to be optimal for targeting the hand motor area. Compared to the sham intervention, the alpha- and beta-tACS resulted in greater alpha and beta oscillations, respectively, while both conditions enhanced cortical inhibition when measured by MEP. The observed effects might be explained by spike-timing-dependent plasticity or the entrainment of intrinsic brain oscillations following tACS.

Furthermore, two studies employed transcranial direct current stimulation (tDCS). In one of them, Kim et al. [5] applied four 20 min sessions of high-definition tDCS at 1 mA to measure different cortical oxyhemoglobin concentrations using functional near-infrared spectroscopy in 26 stroke survivors (one anode and four cathodes, stimulating C3 or C4 according to EEG system for electrode positioning). In this pilot work, oxyhemoglobin concentration only increased in the affected hemisphere, indicating a change in the activity imbalance between the affected and non-affected hemispheres. This could be interpreted as a decrease in motor task-related hemodynamic burden at the level of the affected hemisphere following high-definition tDCS, which might have clinical implications in the rehabilitation field and merits investigation in large-scale controlled studies.

The second study is a case report by Ohnishi et al. [6]. They investigated the effects of 4-week anodal tDCS in addition to gait training in a post-stroke patient (current intensity 2 mA, 20 min sessions, anode 3 cm anterior to Cz EEG electrode position, and cathode over the supraorbital region of the non-injured hemisphere). They found that the bilateral stimulation of supplementary motor areas with gait training led to better electromyographic outcomes (muscle activity and beta intermuscular coherence of the vastus medialis muscle) than gait training alone.

Besides central stimulation techniques, two articles focused on peripheral stimulation. Here, Asao et al. [7] applied a single 20 min session of repetitive peripheral magnetic stimulation ((rPMS), 2 s train per 6 s, 25 Hz) in 14 healthy individuals using a randomized crossover design. They compared the effect of rPMS when it was applied directly over the hand skin or through splint materials (one or two layer(s)). rPMS induced MEP facilitation, which is in line with previous studies on this matter. Some studies have documented rPMS-induced cerebral activation involving the somatosensory, sensorimotor, and frontoparietal regions. The observed rPMS facilitatory effects were only obtained with direct application over the skin but not through splint layers.

Additionally, Von Wrede et al. [8] investigated the daytime-dependent effects of transcutaneous auricular vagus nerve stimulation (taVNS) on EEG recordings in 18 patients being followed at the epileptology department and for whom long-term video EEG recording was needed. In each patient, taVNS was applied in the mid-morning and early afternoon according to the following parameters: biphasic signal applied at 25 Hz, impulse duration of 20 s, and pause of 30 s. They found more considerable network changes after stimulation in the afternoon (i.e., more integrated and less segregated network topology). Therefore, the time of day appears to be a pertinent factor to account for in taVNS studies. Habituation and sham effects could be addressed in the future with double-blind sham-controlled parallel works.

Finally, Kricheldorff et al. [9] present a narrative review on the possibilities of different brain stimulation techniques, i.e., magnetic, electrical, and deep brain stimulation, to modulate cortical neuroplasticity (i.e., MEP, EEG, magnetoencephalography outcomes) and clinical as well as behavioral outcomes. They elegantly tackle the mechanisms of action of these techniques and suggest future paradigms to pave the way for forthcoming research.

## 3. Clinical Outcomes and Neurostimulation

The clinical studies included in this Special Issue mainly focus on central stimulation (one work using tDCS, two works employing rTMS).

Regarding tDCS, in an open-label study, Kurzeck et al. [10] showed that bifrontal tDCS (anode and cathode over F3/F4 EEG electrode positions, respectively) could help improve depression during pregnancy. In this open-label trial, six pregnant women were included. They were treated with a classic tDCS protocol over several weeks (two daily 30 min sessions over ten days followed by the option of one daily 30 min session over ten days). Clinical improvements were observed in terms of the studied outcomes (Hamilton Depression Rating Scale and Beck Depression Inventory), supporting the beneficial effects of this technique in this specific clinical population.

Concerning rTMS, Alhelali et al. [11] investigated the effects of rTMS in a large cohort of patients with unipolar and bipolar depression (*n* = 505). High-frequency rTMS protocols were applied over the left or right dorsolateral prefrontal cortex or the medial prefrontal cortex (mean number of sessions: 18–19). They found that both patient groups demonstrated clinical benefits resulting from rTMS treatment. Still, rTMS is likely more effective in reducing paranoid symptoms in bipolar depression (based on the Hamilton Depression Rating Scale subitem analysis). These interesting findings deserve further confirmation in prospective controlled trials adopting homogenous rTMS protocols.

Additionally, Schoisswohl et al. [12] present data of 22 patients with chronic tinnitus undergoing different rTMS protocols over the temporal cortex (10 sessions, 2000 pulses in total) using personalized (1 vs. 10 vs. 20 Hz; left or right according to parameters with optimal loudness reduction in pre-testing) or standard protocols (1 Hz, left temporal). Although treatment was personalized following pre-testing, the results did not show that personalized treatment was clinically superior over standard treatment (the Tinnitus Functional Index as the primary outcome).

## 4. Conclusions

To sum up, this second Special Issue once again shows the broad application of different central and peripheral nervous system stimulation techniques to modulate neurophysiological or clinical symptoms. Many of the studies are preliminary and are limited by the small sample sizes, the lack of sham groups, or some methodological uncertainties. However, they reflect the potential of different clinical and neurophysiological research applications and point to the further optimization of research questions. More research is needed to further unravel the underlying physiological and biological mechanisms of these techniques and to possibly expand the range of its application alone or in combination with other therapeutic options in neuropsychiatry.
